# Developing Compassionate Community: Insights from the International Standards for Community Development

**DOI:** 10.12688/healthopenres.13611.1

**Published:** 2024-07-12

**Authors:** Wing-Sun Chan, Laura Funk

**Affiliations:** 1 Division of Health Research, Lancaster University, Lancaster, England, LA1 4YX, UK; 2 Department of Sociology and Criminology, University of Manitoba, Winnipeg, Manitoba, Canada

**Keywords:** compassionate community; public health palliative care; health promotion approach; community development; community engagement; community participation; participatory democracy; dying at home in Canada

## Abstract

Aging populations have increased demand for hospice palliative care and support for dying persons. Community support is also becoming an increasingly important aspect of public health intervention. Compassionate communities advocate active bottom-up community participation to strengthen communities’ resilience around death and dying. However, these rapidly growing initiatives face a challenge in putting values such as health equity into practice and in making a social impact through “meaningful participation” at the community level. We reflect on potential challenges related to gendered inequalities, neo-liberal discourses around caring work, and an over-emphasis of community resilience, and argued that these need to be deliberated in compassionate community policy and service development. To address those concerns, in this chapter we argue that the eight components of the International Standards for Community Development Practice or ISCDP (from the International Association for Community Development or IACD) provide important direction about putting values into practice, for instance by advocating for service and policy improvement while engaging in practice and research on compassionate communities. We discuss how the international standards can inform compassionate community development in Canada.

## Introduction

The need for palliative care has been increasing quickly in nations with aging populations, albeit with added complexity during the COVID-19 pandemic ^1^. Yet Allan Kellehear estimates that seriously ill and dying people spend less than 5% of their final year of life in the direct care of health care services, with the vast rest (95%) of their time being supported by non-professional friends and family, or others ^2– 4^. As such, compassionate communities, as a public health palliative care intervention, are growing rapidly worldwide ^5^, inspired by the Ottawa Charter of Health Promotion ^6^ and WHO’s concept of “healthy cities” and “healthy communities.” ^7^ This is a health promotion approach to support solidarity and compassion between community members as people approach the end of their lives ^8^. Taking a socio-ecological perspective, compassionate communities target interactions between environment, policy, community, and individuals to build resilience for supporting people facing death, dying, and grief ^2, 9^. Community engagement, participation, and empowerment are the foundation of building compassionate communities ^10– 12^. As community members reflect on their experiences participating in interventions, this facilitates death literacy: “the knowledge and skills that make it possible to gain access to, understand and act upon end-of-life and death care options” ^13^.

Compassionate community initiatives are being developed in Canada, with organizations like *Pallium Canada* leading the way through projects like their ECHO program ^14– 16^. Canada’s compassionate community approach has been influenced by the Charter of Compassion and the Charter of Compassionate Cities ^17^. Alongside this could be considered school education programs that help teach children how to manage loss including death and bereavement ^18, 19^, campaigns to encourage Canadian workplaces to promote working environments that are responsive to the needs of family carers ^20^, and efforts aligning with Age Friendly and Dementia Friendly Communities that similarly showcase community resilience in supporting care, dying, death, and grieving ^17^. Canadian scholar Mary Lou Kelley’s *Developing Compassionate Community (DCC) Model* (2023) also provides a conceptual guide for those seeking to build community capacity and develop compassionate communities ^21^. The model highlights community development components ranging from situating the problem in the community environment, self-help groups, leadership building, to community-wide participation and action to support people facing death, dying, and grieving ^21^.

Although compassionate community initiatives are growing rapidly in Canada, some groups face greater barriers to accessing support during the dying experience, which reproduces and maintains health inequities ^22, 23^. We therefore need to think about how and whether we might mitigate some potential sources of inequity through community participation ^6^. In this article we cite findings from a recent national research project about dying at home in Canada as well as a review of existing research. We identify how gender inequalities, the neo-liberal discourse of caring work, and an over-emphasis on community resilience in dying at home service and policy initiatives can create particular challenges for compassionate communities’ development. Following this we are exploring the conceptual and practice frames to address health inequity through compassionate community development, drawing inspiration from the International Standards for Community Development Practice (ISCDP) from the International Association for Community Development (IACD).

## Relevant research

A recent mixed methods policy research study in Canada examined public attitudes, qualitative meanings and policy related to dying at home and responsibility for supporting home death. The objectives of the study, broadly, were to examine variation and complexity in Canadians’ attitudes about home death ^24^, as well as variation in qualitative meanings and interpretations of dying at home, including in marginalized or diverse groups ^25^. Our team further explored expectations embedded within policy texts ^26^ with a focus on how policies related to dying at home in Canada can generate inequalities.

The findings from this study and others can help us reflect on compassionate community development in Canada. In particular, we identified three closely inter-related challenges for compassionate community development: gender inequalities ^27^, the neo-liberal discourse associated with caring work, and an over-emphasis on community resilience.

First, the development of compassionate communities in Canada has not comprehensively addressed the gender inequality entailed in the caring work of dying at home. Women often do more, and more intensive forms of caring work when supporting someone to die at home, and this is related to highly gendered moral valuations of care. Females are usually the major caregivers in the end-of-life and palliative care in Canada ^28– 31^. Moreover, as Sutherland and her colleagues point out, common practices in home care nursing can reinforce gendered expectations and exemptions (disadvantaging both men and women) ^30, 31^.

Indeed, women may be the major caregiving force in compassionate communities, not only because of their role in family care provision, but because of their predominance in professional and volunteer roles related to palliative care ^30^. We must directly address the gendered impacts of supporting people who are dying ^31^, while acknowledging important intersectionality related to employment, socio-economic position, age, and gender ^32– 34^. Extending beyond narrow expectations to provide care requires fostering a broader range of ways for those who identify as women to meaningfully participate in building compassionate communities ^35– 37^.

Second, compassionate communities need to be reflexive and aware of the potential for their efforts to reproduce neoliberal discourses of care grounded in narrowly defined concepts of choice, independence, and individual self-responsibility ^38^. For instance, the concepts of choice and informed consent in Canada’s New Medical Assistance in Dying Law (MAiD) has been challenged by several scholars recently as problematic in a context in which it is increasingly difficult to access publicly funded health and social care supports ^39^. Moreover, even preferences for location of death (which Borgstrom identifies that dying at home tends to be used as a crude proxy for ‘choice’ in the end-of-life care policy in England ^38^) are not sole decisions of individuals, but are closely intertwined with perceived availability of and closeness to family and community alongside simultaneous concerns about family well-being ^24, 25^.

Canadians may be reluctant to prefer dying at home in situations involving high symptom severity coupled with low availability of supports; public perceptions in this regard are moreover shaped by orientations towards (or resistance to) the concept of family obligation, as well as experiences of marginalization and structural barriers in the community ^22, 24, 25^. Apart from the symptom management, worries about managing well-being among the dying persons and caregivers can generate significant hesitation ^24, 25^. Such hesitation around dying at home in part signals the importance of a health-promotion model when supporting dying people and their families outside of the context of direct care and healthcare services in Canada ^40, 41^. As a foundational document, The Ottawa Charter for Health Promotion ^6^ invokes the principle of choice, but it does so in a way that foregrounds the social and structural conditions required to allow for choice ^8^.

“…reducing differences in current health status and ensuring equal opportunities and resources to enable all people to achieve their fullest health potential. This includes a secure foundation in a supportive environment, access to information, life skills and opportunities for making healthy choices. People cannot achieve their fullest health potential unless they are able to take control of those things which determine their health. This must apply equally to women and men.” ^6^

Yet in a neoliberal political and economic context, the risks entailed in reproducing the concept or ideal of choice in a narrow way can be particularly pronounced when seeking to promoting community resilience in campaigns and initiatives for compassionate communities. Importantly, a compassionate community not only seeks to develop community resilience in facing death, dying, and grieving, but also focuses on linking dying persons and their families to specialist or generalist palliative care supports and other resources ^2^.

There is a risk that potentially over-emphasizing the importance of the role of community responsibilities may produce an illusion of community empowerment, especially when the initiative or approach places less emphasis on formal palliative and home care supports, for instance ^7, 42^. This can also result in compassionate community initiatives reproducing important geographic inequities when primary responsibility is shifted to communities that face limited access to formal services, such as in rural areas. Indeed when government priorities seek to limit formal service involvement and emphasize self-responsibilities and both self and family care, the compassionate communities movement becomes especially perilous ^43^. There is a related risk that socio-economic and other important inequities can be glossed over or obscured when the term ‘community’ is used. Clear mechanisms to promote inclusive democratic engagement in compassionate communities are essential.

In sum, despite the promise of compassionate community initiatives to enhance the quality of death through mutual support and inter-disciplinary collaboration, developing compassionate communities require mechanisms to promote meaningful participation and collaboration between stakeholders ^9, 12, 44, 45^. The International Standards for Community Development Practice (ISCDP), by the Association for Community Development (IACD) may provide a useful conceptual and practical guides to address the above challenges and promote social justice in the development of a compassionate community.

## Insights from the International Standards for Community Development Practice for compassionate community development

Compassionate communities ^2^ aim at nurturing the civic participation, social solidarity, and empowerment in communities to face death, dying, and grieving ^2, 7– 9, 41, 46^. Although some scholars have developed conceptual guides for the implementation and evaluations of compassionate communities interventions ^12, 40, 44, 47– 49^, there may be additional benefit in linking discussions in this regard to existing international practice standards of community development, as we outline below.

The IACD ^50^ organization was established in 1953 and collaborated during its early years with the United Nations (UN) in a global review emphasizing active community participation in development. Initially focused on developing countries, particularly rural areas, IACD’s scope expanded in the 1960s and ‘70s to include developed countries and anti-poverty programs. The organization underwent reforms in the late 1980s to enhance democratic transparency, and in 1999, IACD was relaunched as a charitable organization. Since then, IACD has expanded its global outreach, forged partnerships with national networks, represented community development at the UN, and started professional development programs. The International Standards for Community Development Practice (ISCDP) were published in 2018 at a conference in Ireland, ^1^ and IACD continues to play a vital role in promoting community development globally, with members from over 70 countries across the world. IACD has a clear definition of community development ^51^:

“Community Development is a practice-based profession and an academic discipline that promotes participative democracy, sustainable development, rights, economic opportunity, equality and social justice, through the organisation, education and empowerment of people within their communities, whether these be of locality, identity or interest, in urban and rural settings (p.13).”

These ISCDP resulted from global exploration of commonalities in community development work worldwide and aimed to assist practitioners and provide support for their endeavors. They comprise eight themes ^52^ which were further elucidated in a 2021 book, *International Community Development Practice:*

1. The ability to apply professional ethics and values in practice
2. The ability to engage with communities
3. The ability to ensure participatory planning
4. The ability to organize for change
5. The ability to support learning for change
6. The ability to promote diversity and inclusion
7. The ability to build leadership and infrastructure
8. The ability to evaluate and improve policy

In what follows, we briefly highlight the content of each theme from the book, tying this to key implications in the context of compassionate community development.

*The ability to apply professional ethics and values in practice* is key practice for social change ^53^. Community development practitioners work with communities for solidarity and agency by facilitating, modelling, practicing, and advocating ^53^. The process is based upon building dialogue, mutual trust and respect between community members and the community development practitioner and agency ^53^.

Community development practitioners thus commit to democratic participation, sustainable development and environmental justice, equality and human rights, social and economic justice, empowerment, and collectively ^54^. Values in community development practice are imperative to resist the “manipulation and dilution” from external influences such as state agencies, funders, politicians and others with their own interests ^55^. Prioritizing values facilitate “conscientization” ^56^ among practitioners and community members, and promotes innovation through exploring new ideas, practices and ways of working, and examining the traditional ways of knowing and doing ^53^.

Compassionate communities aim to promote the well-being of dying individuals and families from a human rights perspective, encompassing physical, psychological, social, and spiritual aspects ^41^. As such, compassionate community development also emphasizes civic participation, in collaboration with mainstream health service ^2^. The ISCDP remind us that the approach is never about top-down service delivery, but a bottom-up equal collaboration between stakeholders between health and non-health sectors ^12^. Stakeholders also need to consider how to ensure that initiatives’ outcomes are closely related to human rights to health.

*The ability to engage with communities* is an essential starting point in community engagement and requires that community development practitioners gather community based knowledge and understanding about problems people are facing, as well as develop social relationships and trust, and nurture collaborative action towards sustainable change ^57^. Importantly, practitioners should not assume community leaders and activists represent the feelings and experiences of all community members ^57^. They also need to strengthen the community development through including community knowledge and skills among the community members ^57^.

Six national community engagement standards identified in this regard include clarity of purpose, commitment to work collaboratively, using a range of suitable engagement methods, clear and regular communication with the stakeholders, identifying and including all the people and organizations related to the issue, and thinking about ways to overcome the barriers of participation ^57, 58^.

As such, the first step to develop compassionate communities is an effective strategy for community engagement ^37^ that includes community members from different backgrounds, to maximize the impact of civic participation in subsequent intervention development and implementation.

*The ability to ensure participatory planning* refers to meaningfully engaging communities in strategic processes and decisions about their future, as equal partnership ^59^. Participatory planning means empowered community stakeholders have the means to influence the actions and outcomes of the decision-making process ^59^. This requires considerations of how and which the stakeholders are involved, as well as barriers and challenges to participation related to power structures ^59^. Achieving and prioritizing bottom-up control is imperative. In this regard, a community development approach to participatory planning should aim at 59:

1. Engaging all appropriate parties in a meaningful manner
2. Being purpose-driven toward an agreed upon goal or set of goals
3. Operating within an agreed upon framework
4. Integrating facts and nurturing ideas
5. Reaching an outcome only after all issues have been fully exhausted

Considering compassionate communities, participatory planning guides us to think about how to help community members achieve a strategic participation process towards a collective decision. This process and decision should align with the value of compassionate community development. Community members may identify barriers, challenges, and power dynamics along the way and practitioners may need to help foster planning, including building up a common framework, meaningful dialogue, reflection ^60^.

*The ability to organize for change* is a crucial process of supporting people to leverage their power and the support of others to improve their situation. Community development practitioners should not just mobilize the community to express their voices, but also find a pathway to reverse power imbalances that hamper their rights and participation in a social system ^61^. 

In terms of practical strategies, community development practitioners may help build an organizational structure that ensures accountability and a sense of collective ownership in the change process ^61^. They can help identify and nurture local leadership and diverse skills and strengths in community members, and to help communities harness their community resources towards improvement ^61^ To maximize change, however, practitioners should themselves build up good local, national, and international networks and partnerships ^61^.

Going beyond a social and health care service, a compassionate community builds a social ecology to support community members facing death, dying, and grieving ^2^. Stakeholders could be both individual community members as well as key organizations, such as schools, shops, and places of worship ^8^. A wide scope is helpful in organizing stakeholders from different parts of community and leverage social capital generated in existing networks to maximize community resilience.

Community development practitioners are required to have professional skills in educational transformation ^62^. The *ability to support learning for change* refers to a “dialogic model” to discover, reflect and create knowledge and skills towards “conscientization” and “praxis” ^63^. This is a way to work towards liberation from existing disempowering social systems and structures and achieve social change ^62^ As such, community development practitioners must convince stakeholders to invest resources and time towards exploring and using and building community experience, knowledge, and skills ^62^. This includes developing learning opportunities that people feel ease with and that they can apply to the action they want to take for change ^62^. Importantly, practitioners should promote learning that reflects the value of community development, such as participatory democracy and social justice ^62^.

Sometimes, community members need help to build additional skills and knowledge to develop compassionate communities, such as death literacy ^10, 13, 64^. In addition, they may need to consider how death literacy can build on and integrate into the tacit knowledge of community members ^65^.

*The ability to promote diversity and inclusion* is vital to community development ^66^. By promoting inclusion, people can participate in the community decision and activities in their own interest ^66^. Inclusion helps all community members, particularly marginalized community members, to share their own lived experience and to engage in social change ^66^. We expect community development practitioners to acquire awareness and understanding about the conditions that shape an excluded group’s experiences ^66^. They also need to ensure engagement, education and organization methods that promote inclusion and respect diversity ^66^. To include different worldviews, practitioners and stakeholders should show cultural respect and ensure safe and inclusive spaces ^66^. The practitioners have a role in helping to resolve conflicts between communities of identity ^66^.

Consistent with the themes of community engagement, participatory planning, and organizing for change, compassionate community builders work with community members towards an agreed upon strategy, mechanism, or framework to promote diversity and inclusion. This is particularly important since marginalized groups are easily excluded from the process of compassionate community development, such as children, seniors, women, sexual minority groups, racialized and immigrant groups, etc. ^22, 29– 31, 34, 67– 70^

*The ability to build leadership and infrastructure* (i.e., organizational and institutional resources) grounded in democratic and participatory practices can foster long-lasting community change and build solidarity and agency within communities ^71^. Helpful community development practices in this regard create a (physical or social) space where people can engage in reciprocal dialogue, learn from each other, and implement and reflect on changes ^72^. Practitioners need to respect group outcomes and action plans while encouraging cycles of practice, action, and thinking ^60, 72^. As such, this principle highlights the importance of community leaders in compassionate community development. One example is compassionate community connectors identified in some research that operate not only as volunteers providing EOL care support but also operate as community brokers ^73– 75^ within informal and formal community support networks ^76, 77^.

*The ability to evaluate and improve policy* speaks to community development practitioners’ mediation role between the people in power and the community. Good evaluation of community development can improve practice towards local, regional, and wider international impact ^78^. It can also leverage community members’ perspectives to ensure they are being heard and systematically responded to ^78^. Community development practitioners, through participatory methods, evaluate progress, present findings back to project stakeholders (including funders), and share them with the community and public policy makers ^78^. As noted in recent discussion of participatory evaluation of the social impact of compassionate community ^5, 79– 81^, it is important to implement creative ways to include evaluation feedback from the community members and systematically disseminate these perspectives ^10^.

**Figure 1.**
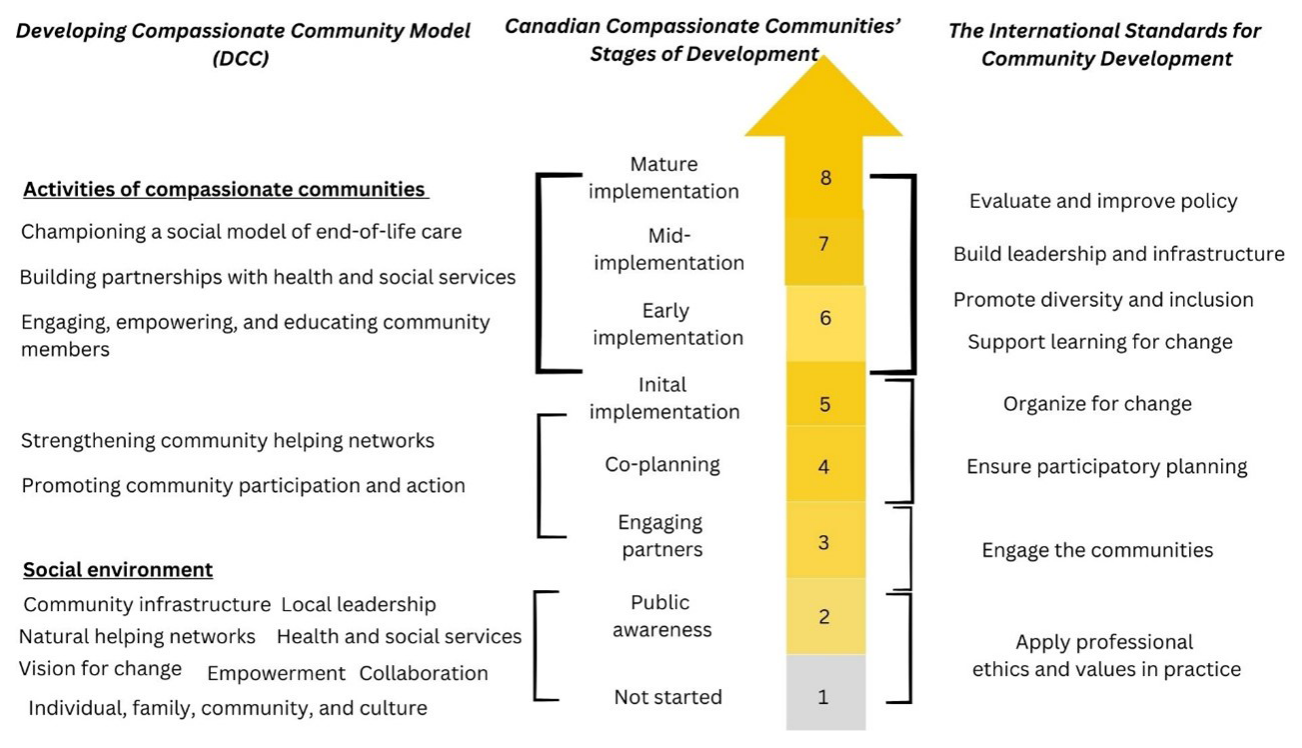
The elements of Canadian Compassionate Community Stages of Development ^84^, Developing Compassionate Community (DCC) model ^21^, and the International Standards for Community Development Practice ^51^.

In sum, the international professional community of community development practitioners have both outlined and provided concrete examples for how community development can embed core values (including those related to participatory democracy and social justice) through practices aimed to organize, educate and empower people, ultimately to achieve bottom-up change and participatory social change. *In essence, we argue that building compassionate communities aligns closely with other community development initiatives.* Therefore, insights from the ISCDP, as outlined above, can supplement other recent conceptual and practical discussions regarding compassionate community development ^10– 12, 44, 48, 49, 73, 82^. Using the ISCDP, compassionate community researchers and practitioners can draw in a fuller and more comprehensive way from the wider practice and knowledge field of community development. Addressing existing challenges, such as gender inequalities, the neoliberal discourse of caring work, and overemphasis on community resilience within compassionate community initiatives in Canada can be facilitated by refining our conceptual model. Including community into the compassionate community development process is key in this regard ^2, 10– 12, 17, 48, 81, 83– 85^. In Figure 1, we compare elements of the ISCDP to current conceptual and practice frames related to compassionate community development in Canada – specifically, to the Canadian Compassionate Community (CCC) Stages of Development ^84^ and Developing Compassionate Community (DCC) model ^21^.

CCC Stages of Development ^84^ identifies several gradual steps towards building compassionate communities, for instance from coordinating a group/organization to start an initiative and raising public awareness ^84^, through engaging potential partners’ and implementation over several years ^84^. Though describing stages of compassionate community development, the model does not provide guidance about how to achieve bottom-up community participation and reduce the risk of reinforcing social inequalities in compassionate community development.

The DCC model makes another helpful contribution to the conceptualization of compassionate community development. It not only outlines stages of development, but discusses key components and activities related to the social environment of initiatives ^21^. It clarifies the role and position of social environment (i.e., the pre-condition of change), community activities (i.e., practical pathway), and service and policy in the development of compassionate communities ^21^.

As outlined above, ISCDP offer guidelines for achieving participatory democracy and social justice in community development; it complements the CCC Stages of Development and DCC models. ISCDP emphasize the importance of values in community development practice, as well as education, inclusion and diversity, leadership, infrastructure, and evaluation ^52^. Integrating the three frames can help compassionate community development practice become more systematic and practical. The models jointly highlight stages, pre-conditions and activities, with implications for building value-driven and inclusive compassionate communities.

## Conclusion

While researchers have identified potential challenges for social equity in compassionate community approaches, we argue that the discussion of compassionate community development from our reflections on the case in Canada could more deeply deliberate conceptual and practice model(s) which help ensure democratic participation towards social justice ^22, 29– 31, 34, 67– 70^. With more sophisticated conceptual and practice model(s), compassionate community initiatives can address the challenges through systematic and sustainable bottom-up community participation. After all, this value was firmly underpinned in the Ottawa Charter of Health Promotion, which is the foundation of the meaningful compassionate community development.

## Data Availability

No data are associated with this article.
