# Developing Compassionate Community: Insights from the International Standards for Community Development

**DOI:** 10.12688/healthopenres.13611.2

**Published:** 2024-09-12

**Authors:** Wing-Sun Chan, Laura Funk

**Affiliations:** 1 Division of Health Research, Lancaster University, Lancaster, England, LA1 4YX, UK; 2 Department of Sociology and Criminology, University of Manitoba, Winnipeg, Manitoba, Canada

**Keywords:** compassionate community; public health palliative care; health promotion approach; community development; community engagement; community participation; participatory democracy; dying at home in Canada

## Abstract

Aging populations have increased demand for hospice palliative care and support for dying persons. More broadly, community support is also becoming an increasingly important aspect of public health intervention. Compassionate communities advocate active bottom-up community participation to strengthen communities’ assets around death and dying. However, these rapidly growing initiatives face a challenge in putting values such as cultural diversity, relationship, and love into practice and in making a social impact through “meaningful participation” at the community level. Reflecting on our experiences in a recent study of dying at home in Canada, we consider potential challenges for compassionate communities more broadly. We argue that risks related to gendered inequity, neo-liberal discourses around caring work, and an over-emphasis of community resilience need to be deliberated in compassionate community policy and service development. To mitigate these risks, we argue that the eight components of the International Standards for Community Development Practice or ISCDP (from the International Association for Community Development or IACD) provide important direction about putting values into practice, for instance by advocating for service and policy improvement while engaging in practice and research on compassionate communities. We discuss how the international standards can inform compassionate community development in Canada.

## Introduction

The need for palliative care has been increasing quickly in nations with aging populations, albeit with added complexity during the COVID-19 pandemic ^1^. Yet Allan Kellehear estimates that seriously ill and dying people spend less than 5% of their final year of life in the direct care of health care services, with the vast rest (95%) of their time being supported by non-professional friends and family, or others ^2– 4^. As such, compassionate communities, as a public health palliative care intervention, are growing rapidly worldwide ^5^, inspired by the Ottawa Charter of Health Promotion ^6^ and WHO’s concept of “healthy cities” and “healthy communities.” ^7^ This is a new public health, asset-based approach that emphasizes solidarity and compassion between community members as people deal with loss and grief, including but not limited to when we approach the end of our lives ^8^. Taking a socio-ecological perspective, compassionate communities target interactions between environment, policy, community, and individuals to activate community assets for supporting people facing death, dying, and grief ^2, 9^. Community engagement, participation, and empowerment are the foundation of building compassionate communities ^10– 12^. As community members reflect on their experiences participating in interventions, this facilitates death literacy: “the knowledge and skills that make it possible to gain access to, understand and act upon end-of-life and death care options” ^13^.

Although distinct from the Charter of Compassion ^14^, compassionate community initiatives in Canada are being developed outside of healthcare settings, with organizations like *Pallium Canada* leading the way through projects like their ECHO program ^15, 16^. Canada’s compassionate community approach has also been tied in important ways to the federal government’s introduction of compassionate care leave benefit provisions in 2004, and modifications of the Criminal Code to legalize Medical Assistance in Dying (MAiD) in 2016 ^17^. Alongside this could be considered school education programs that help teach children how to manage loss including death and bereavement ^18, 19^, campaigns to encourage Canadian workplaces to promote working environments that are responsive to the needs of family carers ^20^, and efforts aligning with Age Friendly and Dementia Friendly Communities that similarly showcase community strengths in supporting care, dying, death, and grieving ^17^. Canadian scholar Mary Lou Kelley’s *Developing Compassionate Community (DCC) Model* (2023) also provides a conceptual guide for those seeking to build on community assets, cultivate inclusivity and diversity, and develop compassionate communities outside of formal healthcare services ^21^. The model highlights civic participation, including situating the problem in the community environment, self-help groups, leadership building, as well as community-wide participation and action to support people facing death, dying, and grieving ^21^.

Although compassionate community initiatives are growing rapidly in Canada, some groups face greater challenges related to dying, which reproduces and maintains unfavorable social determinants ^22, 23^. We therefore need to think about how and whether we might mitigate some potential sources of structural challenges through community participation ^6^. In this article we reflect on the findings from a recent national research project about dying at home in Canada as well as a review of existing research. In a context in which the numbers and proportion of home deaths are increasing, we identify how gender inequities, the neo-liberal discourse of caring work, and an over-emphasis on community resilience in existing service and policy initiatives to promote dying at home can create particular challenges for compassionate communities’ development, while also offering an opportunity for compassionate communities to be part of the solution. This is a starting point for our subsequent exploration of conceptual and practice frames to address various forms of health inequity through compassionate community development, drawing inspiration from the International Standards for Community Development Practice (ISCDP) from the International Association for Community Development (IACD).

## Relevant research

A recent mixed methods policy research study in Canada examined public attitudes, qualitative meanings and policy related to dying at home and responsibility for supporting home death. The objectives of the study, broadly, were to examine variation and complexity in Canadians’ attitudes about home death ^24^, as well as variation in qualitative meanings and interpretations of dying at home, including in marginalized or diverse groups ^25^. Our team further explored expectations embedded within policy texts ^26^ with a focus on how policies related to dying at home in Canada can generate inequities.

The findings from this study and others can help us reflect more broadly on the risks and potential for compassionate community development in Canada, insofar as these findings help direct attention to issues of social structural transformation and structural inequities as well as the broader political-economic context in which compassionate communities unfold. Reflecting on this research project, we inductively identified three closely inter-related structural challenges with implications for compassionate community development: gender inequities ^27^, the neo-liberal discourse associated with caring work, and an over-emphasis on community resilience. Although these challenges may arguably be most prominent in relation to dying at home, this reflection not only illuminates the risks of defining compassionate communities narrowly (i.e., as focused only on dying at home) but highlights systemic issues that may pose similar risks that should be considered in compassionate community development more broadly, especially in areas where there has been expansion of home deaths alongside a growth in neoliberalization.

First, the development of compassionate communities in Canada may encounter the challenge of gender inequity entailed in a broad range of types of relational and caring work, including provisioning, kinkeeping, and emotional labour. Given the focus of our particular project on dying at home, however, we were particularly cognizant that those who identify as female are usually the main caregivers in end-of-life and palliative care in Canada ^28– 31^. Moreover, as Sutherland and her colleagues point out, common practices in home care nursing can reinforce gendered expectations and exemptions in ways that disadvantage both men and women ^30, 31^.

Moreover, it is also possible that women may be the major force in compassionate communities more broadly, and not only because of their role in family care provision or even their predominance in professional and volunteer roles related to palliative care ^30^. Indeed, even aside from their involvement in ‘hands-on’ and intensive forms of care work for dying persons, women often do more to bind families and communities together, and this is related to highly gendered moral valuations of care. We must directly address the potential for broader gendered impacts of supporting dying, grief and loss in care networks and relationships ^31^, while acknowledging other important intersectionalities related to employment, socio-economic position, racialization and age ^32– 34^. Addressing the context of broader gendered power relations is as important to change in this regard as is advocating every community members’ participation in building compassionate communities ^35– 37^. We also need to ensure that community members/practitioners leading compassionate community development initiatives have a strong awareness of gender equity.

Second, compassionate communities in Canada need to be reflexive and aware of the potential for their efforts to reproduce neoliberal discourses of care grounded in narrowly defined concepts of choice, independence, and individual self-responsibility ^38^. For instance, the concepts of choice and informed consent in Canada’s New Medical Assistance in Dying Law (MAiD) have been challenged by several scholars recently as problematic in a context in which it is increasingly difficult to access publicly funded health and social care supports ^39^. Moreover, even preferences for location of death (which Borgstrom identifies that dying at home tends to be used as a crude proxy for ‘choice’ in the end-of-life care policy in England ^38^) are not sole decisions of individuals, but are closely intertwined with perceived availability of and closeness to family and community alongside simultaneous concerns about family well-being ^24, 25^. We need to problematize how MAiD and its underpinning neoliberal rationales can be used in ways that obscure the fundamental components of collective participation (e.g., relationship, affection, and culture) ^8^.

In our recent study, we found that Canadians may be reluctant to prefer dying at home in situations involving high symptom severity coupled with low availability of supports; public perceptions in this regard are moreover shaped by orientations towards (or resistance to) the concept of family obligation, as well as experiences of marginalization and structural barriers in the community ^22, 24, 25^. Apart from symptom management, worries about managing well-being among the dying persons and caregivers can generate significant hesitation ^24, 25^. Such hesitation around dying at home in part signals the importance of a health-promotion model wherein community collectively supports dying people and their families outside of the context of direct care and healthcare services ^40, 41^. As a foundational document, The Ottawa Charter for Health Promotion ^6^ invokes the principle of choice, but it does so in a way that foregrounds the social and structural conditions required to allow for choice ^8^.

“…reducing differences in current health status and ensuring equal opportunities and resources to enable all people to achieve their fullest health potential. This includes a secure foundation in a supportive environment, access to information, life skills and opportunities for making healthy choices. People cannot achieve their fullest health potential unless they are able to take control of those things which determine their health. This must apply equally to women and men.” ^6^

Yet in a neoliberal political and economic context, the risks of reproducing the concept or ideal of choice in a narrow way can be particularly pronounced not only in an over-emphasis on dying at home, but in campaigns and initiatives for compassionate communities that centre around “community resilience” as a major outcome. Importantly, a compassionate community does not seek to develop community resilience (i.e., the deficit model) in facing death, dying, and grieving, but focuses on linking dying persons and their families to the community ecology (i.e., the asset model) ^2^ and on recognizing and developing more nuanced and complex experiences of suffering.

Indeed, the disconnect between palliative care service provision and policy and compassionate communities generates the possibility of over-emphasizing the role of community responsibilities and thus producing an illusion of community empowerment, especially if existing initiatives become coopted for government goals or place less emphasis on formal social and care supports, for instance ^7, 42^. This can also result in compassionate community initiatives reproducing important geographic inequities when primary responsibility is shifted to communities that face limited access to formal services, such as in rural areas. Indeed, in a context in which government priorities seek to limit formal service involvement and emphasize self-responsibilities and both self and family care, the compassionate communities movement can become obscured in important ways ^43^. There is a related risk that socio-economic and other important inequities can be glossed over or obscured when the term ‘community’ is used. To help address such challenges, palliative care provision needs to be fundamentally reoriented from a biomedically focused mechanic service delivery model to an organic participatory collaboration model (between generalist palliative care, specialist palliative, compassionate communities, and civic participation), as reflected in the Compassionate City Charter ^2^.

In sum, although compassionate community initiatives are promising for enhancing the quality of death, mutual support, and civic participation, their development requires social solutions and meaningful participation and collaboration between community stakeholders ^9, 12, 44, 45^. This community-based participation aspect that can help shift the imagination of service and policy sectors is beyond a dominant of biomedical, clinical, and personal service-oriented interventions, such as palliative, end-of-life and hospice care, dying at home, and advance care planning. The International Standards for Community Development Practice (ISCDP), by the Association for Community Development (IACD) may provide a useful conceptual and practical guide to address the potential challenges outlined above, and promote social justice in the development of a compassionate community.

## Insights from the International Standards for Community Development Practice for compassionate community development

Compassionate communities ^2^ aim at nurturing the civic participation, social solidarity, and empowerment in communities to face death, dying, and grieving ^2, 7– 9, 41, 46^. Although some scholars have developed conceptual guides for the implementation and evaluations of compassionate communities interventions ^12, 40, 44, 47– 49^, there may be additional benefit in linking discussions in this regard to existing international practice standards of community development, as we outline below. The discussion also informs how to address the potential risks of reproducing gender inequalities, neo-liberal discourse associated with caring, and over-emphasis on resilience, when developing compassionate communities within neoliberal contexts that increasingly emphasize dying at home.

The IACD ^50^ organization was established in 1953 and collaborated during its early years with the United Nations (UN) in a global review emphasizing active community participation in development. Initially focused on developing countries, particularly rural areas, IACD’s scope expanded in the 1960s and ‘70s to include developed countries and anti-poverty programs. The organization underwent reforms in the late 1980s to enhance democratic transparency, and in 1999, IACD was relaunched as a charitable organization. Since then, IACD has expanded its global outreach, forged partnerships with national networks, represented community development at the UN, and started professional development programs. The International Standards for Community Development Practice (ISCDP) were published in 2018 at a conference in Ireland, ^1^ and IACD continues to play a vital role in promoting community development globally, with members from over 70 countries across the world. IACD has a clear definition of community development ^51^:

“Community Development is a practice-based profession and an academic discipline that promotes participative democracy, sustainable development, rights, economic opportunity, equality and social justice, through the organisation, education and empowerment of people within their communities, whether these be of locality, identity or interest, in urban and rural settings (p.13).”

These ISCDP resulted from global exploration of commonalities in community development work worldwide and aimed to assist practitioners and provide support for their endeavors. They comprise eight themes ^52^ which were further elucidated in a 2021 book, *International Community Development Practice:*

1. The ability to apply professional ethics and values in practice
2. The ability to engage with communities
3. The ability to ensure participatory planning
4. The ability to organize for change
5. The ability to support learning for change
6. The ability to promote diversity and inclusion
7. The ability to build leadership and infrastructure
8. The ability to evaluate and improve policy

In what follows, we briefly highlight the content of each theme from the book, tying this to key implications in the context of compassionate community development.

*The ability to apply professional ethics and values in practice* is key for social change ^53^. Community development practitioners work with communities for solidarity and agency by facilitating, modelling, practicing, and advocating ^53^. The process is based on building dialogue, mutual trust and respect between community members and community development practitioners and agencies ^53^.

Community development practitioners thus commit to democratic participation, sustainable development and environmental justice, equality and human rights, social and economic justice, empowerment, and collectiviy ^54^. Values in community development practice are imperative to resist the “manipulation and dilution” from external influences such as state agencies, funders, politicians and others with their own interests ^55^. Prioritizing values facilitate “conscientization” ^56^ among practitioners and community members, and promotes innovation through exploring new ideas, practices and ways of working, and examining traditional ways of knowing and doing ^53^.

Compassionate communities aim to promote the well-being of dying individuals and families from a human rights perspective, encompassing physical, psychological, social, and spiritual aspects ^41^. As such, compassionate community development also emphasizes civic participation ^2^. The ISCDP reminds us that the approach is never about top-down social solutions, but a bottom-up equal collaboration between community stakeholders ^12^. Stakeholders also need to consider how to ensure that initiatives’ outcomes are closely related to human rights and to health. For example, prior to any interventions, compassionate community organizers, should develop knowledge and awareness that would assist them in identifying and mitigating gender inequities in compassionate community work, as well as knowledge and awareness of how neo-liberal discourse operates and can be resisted in compassionate community development (e.g., including the risk of over-emphasis on community resilience).

*The ability to engage with communities* is an essential starting point in community engagement and requires that community development practitioners gather community-based knowledge and understanding about problems people are facing, as well as develop social relationships and trust, and nurture collaborative action towards sustainable change ^57^. Importantly, practitioners should not assume community leaders and activists represent the feelings and experiences of all community members ^57^. They also need to strengthen and include knowledge and skills among a broader range of community members ^57^.

Six national community engagement standards identified in this regard include clarity of purpose, commitment to work collaboratively, using a range of suitable engagement methods, clear and regular communication with the stakeholders, identifying and including all the people and organizations related to the issue, and thinking about ways to overcome barriers to participation ^57, 58^.

As such, the first step to develop compassionate communities is an effective strategy for community engagement ^37^ that includes community members from different backgrounds, to maximize the impact of civic participation in subsequent intervention development and implementation. For example, organizers should reflect on an engagement strategy to connect with women and caregivers, as well as broader community members.

*The ability to ensure participatory planning* refers to meaningfully engaging communities in strategic processes and decisions about their future, as equal partnership ^59^. Participatory planning means empowered community stakeholders have the means to influence the actions and outcomes of the decision-making process ^59^. This requires considerations of how and which stakeholders are involved, as well as barriers and challenges to participation related to power structures ^59^. Achieving and prioritizing bottom-up control is imperative. In this regard, a community development approach to participatory planning should aim at ^59^:

1. Engaging all appropriate parties in a meaningful manner
2. Being purpose-driven toward an agreed upon goal or set of goals
3. Operating within an agreed upon framework
4. Integrating facts and nurturing ideas
5. Reaching an outcome only after all issues have been fully exhausted

Considering compassionate communities, participatory planning guides us to think about how to help community members achieve a strategic participation process towards a collective decision. This process and decision should align with the value of compassionate community development. Community members may identify barriers, challenges, and power dynamics along the way and practitioners may need to help foster planning, including problem posing, building up a common analytic framework, meaningful dialogue, and reflection ^60^. For example, organizers should ensure women and caregivers, as well as broader community members can fully participate in different development stages while having the ability to successfully articulate their concerns. This requires, in turn, that compassionate community organizers are open to and welcoming of critique.

*The ability to organize for change* is a crucial process of supporting people to leverage their power and the support of others to improve their situation. Community development practitioners should not just mobilize the community to express their voices, but also find a pathway to reverse power imbalances that hamper their rights and participation in a social system ^61^. 

In terms of practical strategies, community development practitioners may help build an organizational structure that ensures accountability and a sense of collective ownership in the change process ^61^. They can help identify and nurture local leadership and diverse skills and strengths in community members, and to help communities harness their community resources towards improvement ^61^. To maximize change, however, practitioners should themselves build up good local, national, and international networks and partnerships ^61^.

Going beyond a social and health care service perspective, a compassionate community builds a broader social and relational ecology to support community members facing death, dying, and grieving ^2^. Stakeholders could be both individual community members as well as key organizations, such as schools, shops, and places of worship ^8^. A wide scope is helpful in organizing stakeholders from different parts of community and leveraging social capital generated in existing networks to maximize community resilience. For example, organizers should ensure a participatory organizational and governance structure for compassionate community development, which includes different stakeholders.

Community development practitioners are required to have professional skills in educational transformation ^62^. The *ability to support learning for change* refers to a “dialogic model” to discover, reflect and create knowledge and skills towards “conscientization” and “praxis” ^63^. This is a way to work towards liberation from existing disempowering social systems and structures and achieve social change ^62^ As such, community development practitioners must convince stakeholders to invest resources and time towards exploring and using and building community experience, knowledge, and skills ^62^. This includes developing learning opportunities that people feel at ease with and that they can apply to the action they want to take for change ^62^. Importantly, practitioners should promote learning that reflects the value of community development, such as participatory democracy and social justice ^62^.

Sometimes, community members need help to build additional skills and knowledge to develop compassionate communities, such as death literacy ^10, 13, 64^. In addition, they may need to consider how death literacy can build on and integrate tacit community knowledge ^65^. Organizers should consider training opportunities for women, caregivers, and broader community members to help them gain meaningful and relevant practical community development and organizing skills, as well as social justice awareness and knowledge.

*The ability to promote diversity and inclusion* is vital to community development ^66^. By promoting inclusion, people can participate in community decisions and activities in their own interest ^66^. Inclusion helps all community members, particularly marginalized community members, to share their own lived experience and to engage in social change ^66^. We expect community development practitioners to acquire awareness and understanding about the conditions that shape an excluded group’s experiences ^66^. They also need to ensure engagement, education and organization methods that promote inclusion and respect diversity ^66^. To include different worldviews, practitioners and stakeholders should show cultural respect and ensure safe and inclusive spaces ^66^. These practitioners also have a role in helping to resolve conflicts between communities of identity ^66^.

Consistent with the themes of community engagement, participatory planning, and organizing for change, compassionate community builders work with community members towards an agreed upon strategy, mechanism, or framework to promote diversity and inclusion. This is particularly important since marginalized groups are easily excluded from the process of compassionate community development, such as children, seniors, women, sexual minority groups, racialized and immigrant groups, etc. ^22, 29– 31, 34, 67– 70^ Organizers should have a high awareness of the challenges and barriers to diversity and inclusiveness within compassionate community development; in turn, they should aim to discuss such issues at the level of the initiative’s organizational and governance structure.

*The ability to build leadership and infrastructure* (i.e., organizational and institutional resources) grounded in democratic and participatory practices can foster long-lasting community change and build solidarity and agency within communities ^71^. Helpful community development practices in this regard create a (physical or social) space where people can engage in reciprocal dialogue, learn from each other, and implement and reflect on changes ^72^. Practitioners need to respect group outcomes and action plans while encouraging cycles of practice, action, and thinking ^60, 72^. As such, this principle highlights the importance of community leaders in compassionate community development. One example is compassionate community connectors identified in some research that operate not only as volunteers providing EOL care support but also operate as community brokers ^73– 75^ within informal and formal community support networks ^76, 77^. Organizers should also ensure that women, caregivers, and other community stakeholders can be in different leadership positions at different levels throughout the development process.

*The ability to evaluate and improve policy* speaks to community development practitioners’ mediation role between the people in power and the community. Good evaluation of community development can improve practice towards local, regional, and wider international impact ^78^. It can also leverage community members’ perspectives to ensure they are being heard and systematically responded to ^78^. Community development practitioners, through participatory methods, evaluate progress, present findings back to project stakeholders (including funders), and share them with the community and public policy makers ^78^. As noted in recent discussion of participatory evaluation of the social impact of compassionate community ^5, 79– 81^, it is important to implement creative ways to include evaluation feedback from the community members and systematically disseminate these perspectives ^10^. Practitioners should work with community members to advocate for the evaluation and transformation of policy for compassionate community development. In this regard they can ally and connect with academics and political leaders to raise issues of gender inequities, neo-liberal discourse associated with caring, and over-emphasis on resilience in relation to existing policy and practice emphases on dying at home; in turn, they can work to shift policy conversations towards more broadly defined compassionate communities, that can help mitigate these challenges.

In sum, the international professional community of community development practitioners have both outlined and provided concrete examples for how community development can embed core values (including those related to participatory democracy and social justice) through practices aimed to organize, educate and empower people, ultimately to achieve bottom-up change and participatory social change. *In essence, we argue that building compassionate communities aligns closely with other community development initiatives.* Therefore, insights from the ISCDP, as outlined above, can supplement other recent conceptual and practical discussions regarding compassionate community development ^10– 12, 44, 48, 49, 73, 82^. Using the ISCDP, compassionate community researchers and practitioners can draw in a fuller and more comprehensive way from the wider practice and knowledge field of community development. Addressing existing challenges, such as gender inequities, the neoliberal discourse of caring work, and the risk of overemphasizing community resilience within compassionate community initiatives in Canada can be facilitated by refining our conceptual model. Including community into the compassionate community development process is key in this regard ^2, 10– 12, 17, 48, 81, 83– 85^. We examine elements of the ISCDP to current conceptual and practice frames related to compassionate community development in Canada – specifically, to the Canadian Compassionate Community (CCC) Stages of Development ^84^ and Developing Compassionate Community (DCC) model ^21^.

CCC Stages of Development ^84^ identifies several gradual steps towards building compassionate communities, for instance from coordinating a group/organization to starting an initiative and raising public awareness ^84^, through engaging potential partners and implementation over several years ^84^. Though describing stages of compassionate community development, the model provides guidance for achieving bottom-up community participation and reducing the risk of reinforcing social inequalities in compassionate community development.

The DCC model makes another helpful contribution to the conceptualization of compassionate community development. It not only outlines stages of development, but discusses key components and activities related to the social environment of initiatives ^21^. It clarifies the role and position of social environment (i.e., the pre-condition of change), community activities (i.e., practical pathway), and service and policy in the development of compassionate communities ^21^.

As outlined above, ISCDP offer guidelines for achieving participatory democracy and social justice in community development; it complements the CCC Stages of Development and DCC models. ISCDP emphasize the importance of values in community development practice, as well as promoting inclusion and diversity ^52^. Education, leadership, infrastructure, and evaluation are necessary action points in community development ^52^. The three frames can help manifest compassionate community development practice that is iterative and cyclical. The models jointly highlight values, stages, pre-conditions and activities, with implications for building value-driven and inclusive compassionate communities.

## Conclusion

Though grounded in research specific to dying at home, we have identified potential challenges related to social equity that we argue should also be considered as potential risks within compassionate community development, especially if the implementation of the latter is overly narrow in focus or breadth. A broad conceptualization of compassionate communities is necessary but not sufficient to fully mitigate these risks, however. We argue that discussions of compassionate community development could more deeply deliberate conceptual and practice model(s) which help ensure democratic participation towards social justice ^22, 29– 31, 34, 67– 70^. With more sophisticated conceptual models of practice, compassionate community initiatives can better address potential challenges related to the broader context in which they are implemented, as they work towards reorienting palliative care services towards a broader social ecology, community asset perspective, and sustainable organic participatory collaboration.

## Data Availability

No data are associated with this article.
