# Supplementary material for: Developing Compassionate Community: Insights from the International Standards for Community Development
Source: Health Open Res. 2024 Sep 12;6:17. Originally published 2024 Jul 12. [Version 2] doi: 10.12688/healthopenres.13611.2 (PMC12368493; doi:10.12688/healthopenres.13611.2)
Supplement: Peer review history

# Reviewer report: Reviewer response for version 2

**DOI:** 10.21956/healthopenres.14870.r29287

**Published:** 2025-08-20

**Authors:** Sandy Whitelaw

**Affiliations:** 1 School of Social and Environmental Sustainability/Glasgow End of Life Studies Group, University of Glasgow, Dumfries, Scotland, UK

First, I note the existence of significant, in-depth and informed review critique and that the authors have made significant changes to earlier drafts in relation to comments.  

 As other reviewers have suggested, I think the general orientation of the paper towards realistic implementation (and the acceptance of potential challenges) is hugely helpful in the Compassionate Communities domain. I see this as constructive in translating the undoubtedly positive CC values into actual practice.

 This work is well contextualised in relevant Canadian policy and research on approaches to the end of life. The surfacing of a series of *potential* issues (e.g. gender and geographic inequity in care and the dangers of excessive ‘responsibalisation’ with an associated drift towards neo-liberal policy ideology) can only be constructive. I note that some other reviewers have questioned whether these issues are *necessarily* present within Compassionate Communities specifically, but given their gravity, I see no reason why the possibility of such trends should not be critically considered.     

 The application of an actual framework to achieve this is to my eyes innovative and fills a gap in the Compassionate Communities literature in relation to the depth of critical evaluative reflection. The components of the International Standards for Community Development Practice or ISCDP (from the International Association for Community Development or IACD) appear highly relevant. As well as general principles of project governance and evaluation, to my eyes, the themes exhibit insights into two particularly important issues to Compassionate Communities: professional ethics and values and the nature of ‘participation’ (and associated power relations between professionals and civic communities).

 I note the critique that Compassionate Communities are not simply about care – but I see no reason why these generalised themes could not be applied to a wider range of non-care based ‘community’ and ‘civic’ activities associated with Compassionate Communities. I note that some reviewers have suggested that the paper needs to make it specifically clear that this work relates to the *care* aspect of Compassionate Communities (which would be useful). However, depending on the views of the authors, it could also perhaps be recognised that the themes might be applied to all professional-community interactions and reflected/acknowledged in the paper?

 So in summary, given the extent of previous comment and associated responses from the authors, I wouldn’t see any benefit in suggestions for further amendments. I would see the paper as it stands as a very useful foundation for (hopefully) subsequent application in an empirical/realistic context.

Is the topic of the opinion article discussed accurately in the context of the current literature?

Yes

Are arguments sufficiently supported by evidence from the published literature?

Yes

Are all factual statements correct and adequately supported by citations?

Yes

Are the conclusions drawn balanced and justified on the basis of the presented arguments?

Yes

Reviewer Expertise:

Public Health and end of Life Policy; specifically the settings based element of the Ottawa Charter

I confirm that I have read this submission and believe that I have an appropriate level of expertise to confirm that it is of an acceptable scientific standard.

# Reviewer report: Reviewer response for version 2

**DOI:** 10.21956/healthopenres.14870.r29178

**Published:** 2025-02-10

**Authors:** Katharina Heimerl

**Affiliations:** 1 University Vienna, Vienna, Vienna, Austria

The insight that the Compassionate Community movement is not sufficiently linked to the knowledge and experiences of Community Development is compelling and the authors demonstrate this very clearly in the opinion paper. To bring together these two traditions is a real gain for both sides. Moreover, the challenges of Compassionate Communities, consisting above all in gender inequities, neoliberal discourse around care work and over-emphasis of community resilience, are well elaborated.

 Nevertheless, I would like to address a few minor changes that would benefit the paper. The changes relate primarily to the structure of the paper.

 In the first sentence you connect the increasing need for palliative care with the aging of the population. This requires further explanation: In my view, the aging of a person or a population per se does not result in an increased need for palliative care.

 The second sentence starts with “Yet Allan Kellehear…” whereas none of the mentioned references is attributable to Kellehear alone.

 The paragraph “Relevant research” deals with three major challenges or even risks of Compassionate Communities. The heading should reflect this, i.e. I suggest to replace “Relevant research” by “Challenges and risks of Compassionate Communities” or similar. I would also suggest introducing subheadings for each of the three challenges.

 In the next paragraph you show, in what ways the eight themes of ISCDP may add to the development of Compassionate Communities. This goes far beyond mitigating the three challenges defined in the introduction. Therefore, the two parts of the paper “Relevant research” and “Insights form ISCDP” do not seem to be sufficiently connected to each other. On page 5 you state: “In what follows, we briefly highlight the content of each theme from the book, tying this to key implications in the context of compassionate community development”. This sounds like the overall aim of your paper and I would suggest, you introduce this aim in the last paragraph of the introduction. In what way do the three challenges that you identified in the beginning of the text tie in here? Are the “key implications in the context of compassionate community development” identical to the three challenges? This needs to be specified in the introduction.

 I recommend to start the chapter “Conclusion” (i.e. to simply move the header) with page 5, last paragraph starting with “In sum….”

 The twofold aim of the paper (to discuss the three challenges and to demonstrate the CCC would benefit from integrating ISCDP) persists also in the conclusion and should be merged.

 I would be very interested to understand, whether ICDSP can benefit from the experiences of CCC, can you add a sentence about it in the conclusion?

Is the topic of the opinion article discussed accurately in the context of the current literature?

Yes

Are arguments sufficiently supported by evidence from the published literature?

Yes

Are all factual statements correct and adequately supported by citations?

Yes

Are the conclusions drawn balanced and justified on the basis of the presented arguments?

Yes

Reviewer Expertise:

Palliative Care, Palliative Geriatrics, Participatory Research, Caring Community

I confirm that I have read this submission and believe that I have an appropriate level of expertise to confirm that it is of an acceptable scientific standard, however I have significant reservations, as outlined above.

# Reviewer report: Reviewer response for version 2

**DOI:** 10.21956/healthopenres.14870.r29180

**Published:** 2024-12-24

**Authors:** John Patrick Rosenberg

**Affiliations:** 1 University of the Sunshine Coast, Sippy Downs, Queensland, Australia

In light of the four reviews already in place, I've fashioned my feedback as commentary for further consideration rather than specific amendments to the current version. 

 This article offers a useful commentary on the complex interfaces between Compassionate Communities, Community Development, and dying at home. Importantly, it differentiates between the community as a setting of care (i.e.: dying at home) and communities whose collective capacity to engage in matters of caregiving, dying, loss and grief form a social approach to these issues.

 Read through this lens, it can be seen to be embedded in a specific context (Canada) for a specific group (those caregiving at home). Extrapolating beyond these contextual parameters remains an important part of the evolution of thinking in Compassionate Communities and Public Health Palliative Care. This includes mention of the Charter of Compassion – although not globally adopted, it remains part of the broader discourse of compassion; for example, a city that is compassionate about dying can be compassionate about homelessness, refugees and other examples of social disadvantage. There is a valid justification to explore this nexus more fully in future work, given that the distinctions made between the frameworks towards compassionate actions in communities may not be as much a concern to citizens as they are to their apologists.

 I share some other reviewers’ reservations about the assumptions regarding gendered inequity and caregiving work. It is certainly a known feature of care in the home setting, but is it reflective of a broader social approach? Is the determination of participation in the social world itself gendered? This article asks whether women ‘may be the major force in compassionate communities more broadly’. For this to be ‘measured’, an examination of the *nature* of participation is as important as the people participating.

 Conceptual mapping is a useful feature of evolving conceptualisation and like any aspect of the social world, is dynamic over time.  This article provides some preliminary consideration of how Compassionate Communities might map against other frameworks; further exploration would be valuable.

 Overall, I found the article stimulating and worthwhile to provoke thinking about Compassionate Communities.

Is the topic of the opinion article discussed accurately in the context of the current literature?

Yes

Are arguments sufficiently supported by evidence from the published literature?

Partly

Are all factual statements correct and adequately supported by citations?

Partly

Are the conclusions drawn balanced and justified on the basis of the presented arguments?

Partly

Reviewer Expertise:

Health Promoting Palliative Care; Public Health Palliative Care; community practice; Compassionate Communities; Death Literacy.

I confirm that I have read this submission and believe that I have an appropriate level of expertise to confirm that it is of an acceptable scientific standard, however I have significant reservations, as outlined above.

# Reviewer report: Reviewer response for version 2

**DOI:** 10.21956/healthopenres.14870.r29071

**Published:** 2024-11-28

**Authors:** Sashiprabha Dulanjalee Nawaratne

**Affiliations:** 1 Directorate of Healthcare Quality and Safety, Colombo, Sri Lanka

I appreciate the opportunity to review this opinion article on 'Developing Compassionate Community: Insights from the International Standards for Community Development'.  It effectively raises critical questions and thoughtfully applies the ISCDP framework to compassionate communities.

 Overall, the article is commendably well-written and the latter part of the article is quite clear and aligns well with the objectives the authors have mentioned.

 However, up to that point, there are some areas that need clarification.

 In the Introduction; The Charter for Compassion - Are you referring to the Compassionate City Charter? It is my understanding that the public health approach to palliative care incorporates both top-down strategies, such as compassionate cities, and bottom-up initiatives, like compassionate communities. However, the document you have cited may not accurately reflect this charter. 

 I felt that if the authors had included their argument mentioned under 'recent research'  across the areas of ISCDP it would help to understand the article better as that seems to be the overall aim and is reflected in the title. Some of the challenges mentioned e.g dying at home, and gender equity may be relevant to several areas of the ISCDP. 

 I wanted to clarify that my comments and suggestions are purely aimed at understanding the article better and enhancing its overall quality. I appreciate the effort and thought that has gone into this article, and I believe that addressing the points mentioned above could further strengthen its impact.

Is the topic of the opinion article discussed accurately in the context of the current literature?

Yes

Are arguments sufficiently supported by evidence from the published literature?

Partly

Are all factual statements correct and adequately supported by citations?

Partly

Are the conclusions drawn balanced and justified on the basis of the presented arguments?

Yes

Reviewer Expertise:

public health, palliative and end-of-life care

I confirm that I have read this submission and believe that I have an appropriate level of expertise to confirm that it is of an acceptable scientific standard, however I have significant reservations, as outlined above.

# Reviewer report: Reviewer response for version 2

**DOI:** 10.21956/healthopenres.14870.r28818

**Published:** 2024-11-06

**Authors:** Julian Abel

**Affiliations:** 1 Compassionate Communities UK, St. Martin, Helston, UK

The paper is much improved from the original version. There is however still a flaw that needs addressing, in addition to the comments from the other reviewers. The paper argues that gender inequality with regards to caring is a problem and that neo liberal discourses are at risk of worsening this. Thisi s a very specific argument which applies to small parts of compassionate communities - direct care of someone who is dying and home deaths.

 Compassionate communities is a phrase which refers to a much bigger way of approaching death, dying, loss and care giving. It is not just about hands on care, and not just about home death. It is the web of interrelationships between all involved - how people can support each other.

 Everyone has a part of to play in enhancing love, laughter and friendship as well as the practical tasks. Death literacy lies at the core. The paper argues that the IACD guidelines should be applied generally to resolve the issues outlined, yet the analysis refers to the smaller subset of home dying and hands on caring.  There is no doubt that the IACD guidelines are a helpful checklist for community development practitioners, including those involved in compassionate communities at end of life.

 The paper would benefit from reframing the argument to limit itself to comments on hands on care and home dying, rather than try and cover the whole approach to compassionate communities, because their evidence is confined to this part of the analysis. It is sensible to suggest that those involved in compassionate communities use the IACD guidelines to ensure their practice is sufficient but this is not an argument to be made as a main part of the paper.  There are a couple of other smaller points. There is no need to mention the Charter for Compassion. This is introduced by the authors but is not part of compassionate communities at end of life. In addition in paragraph 3 the phrase we is used a number of times but it is unclear as to who the authors are referring.

Reviewer Expertise:

Public health palliative care

I confirm that I have read this submission and believe that I have an appropriate level of expertise to confirm that it is of an acceptable scientific standard, however I have significant reservations, as outlined above.

# Reviewer report: Reviewer response for version 2

**DOI:** 10.21956/healthopenres.14870.r28874

**Published:** 2024-10-17

**Authors:** Émilie Lessard

**Affiliations:** 1 Centre de Recherche du Centre Hospitalier de l’Université de Montréal, Montréal, Canada

**Introduction** In my view, the compassionate communities movement or community engagement is not a public health "intervention", but a social and community-based approach to death and dying. 

 The paper could benefit from a more precise and consistent definition of key terms (there is a lot of confusion among them). For example, the introduction mentions "compassionate communities" as a public health intervention, but later suggests it's a broader social approach. Providing a clear, distinct and concise definition of "compassionate communities," "civic engagement," and "community engagement" early on would improve clarity and consistency throughout the paper. Also, while you refer to compassionate communities, the examples provided are not community-based initiatives (e.g. national campaigns, school education programs, workplaces). 

 Is there really a distinct Canadian compassionate community approach (what would it be)?

 Additionally, the connection between compassionate communities and the examples provided (e.g., MAiD, compassionate care leave, national campaigns) could be more clearly articulated. For instance, the authors could explore how these policies and initiatives might support or hinder the development of compassionate communities, or how compassionate communities might influence these policies and initiatives.

**Relevant Research** You seem to be confusing the issues of dying at home, public policies and the burden on women caregivers with those of compassionate community development. You have no data to infer that these issues are the same within the compassionate community movement (that goes beyond dying at home). While the paper identifies three structural challenges (gender inequities, neoliberal discourse, and over-emphasis on community resilience), it would be beneficial to provide more context and evidence for how these challenges specifically manifest within the compassionate community development.

 For example, the authors mention that women are often the primary caregivers in end-of-life care. To strengthen this point, they could provide data on the proportion of women involved in compassionate community initiatives, or discuss how gendered expectations might impact the types of roles women occupy within these initiatives. Similarly, when discussing the neoliberal discourse of care, the authors could provide specific examples of how this discourse is reflected in compassionate community initiatives or policies. They could also explore how compassionate communities might actively challenge or resist this discourse. As it stands, your article does not allow us to draw such a conclusion.

**Insights from the International Standards for Community Development Practice**:

 The section on the International Standards for Community Development Practice (ISCDP) is comprehensive but could be streamlined to focus on the most relevant themes for compassionate communities. For example, the themes of "promoting diversity and inclusion" and "building leadership and infrastructure" seem particularly relevant. The authors could provide more specific examples of how these themes apply to compassionate communities, such as discussing how to ensure the inclusion of marginalized groups or how to develop sustainable leadership structures within these initiatives.

 The goal of public health approaches to death and dying, from which compassionate community are the best-known example, is to act on social determinants of death, dying and grief, provide a social model of PCEoL care, and to reduce social inequalities by promoting equity and inclusion.

 I don't see the relevance of the aforementioned standards, as compassionate community has already been thoroughly conceptualized in the past 20 years (as it is the case for other social innovations using community development).  

 The paper does not acknowledge that compassionate communities have been extensively conceptualized in the past 20 years. To further highlight the contribution of this paper, the authors could more explicitly articulate the specific gap they are addressing and how their analysis adds to the existing literature. They could also emphasize the unique aspects of their approach, such as the focus on structural challenges and the application of the ISCDP framework.

**Conclusion** The conclusion could be strengthened by providing a more concrete and actionable call to action. What specific steps can be taken to promote the development of equitable and inclusive compassionate communities? How can policymakers, healthcare providers, and community members work together to address the identified challenges?

Is the topic of the opinion article discussed accurately in the context of the current literature?

No

Are arguments sufficiently supported by evidence from the published literature?

No

Are all factual statements correct and adequately supported by citations?

No

Are the conclusions drawn balanced and justified on the basis of the presented arguments?

Partly

Reviewer Expertise:

Community engagement in public health approaches to death and dying; compassionate community; anthropology of death; palliative home care and home death.

I confirm that I have read this submission and believe that I have an appropriate level of expertise to state that I do not consider it to be of an acceptable scientific standard, for reasons outlined above.

# Reviewer report: Reviewer response for version 2

**DOI:** 10.21956/healthopenres.14870.r28817

**Published:** 2024-10-03

**Authors:** Steven Vanderstichelen

**Affiliations:** 1 Ghent University, Vrije Universiteit Brussel, Brussels, Belgium

I thank the authors for their work revising the paper and responding to each of my comments. I only have a few minor comments remaining.

**Minor comments**

 “Although distinct from the Charter of Compassion 14, compassionate community initiatives in Canada are being developed outside of healthcare settings, with organizations like *Pallium Canada* leading the way through projects like their ECHO program 15, 16.”

- The use of ‘although’ creates the impression that an argument is being made but it’s not explicit. Now it implies that a comparison is being made between the charter for compassion and the compassionate cities charter? or the compassionate communities movement? Is the argument that the charter for compassion also argues for initiatives to be developed specifically outside of healthcare settings? It seems like a thin comparison and I wonder if it’s necessary. I understand the authors may want to acknowledge the charter for compassion but the paper would benefit from a more meaningful and explicit comparison in this case.

The authors have now added more examples of how the ISCDP address the critical issues identified by the authors at the beginning of the paper. However I think the text could use more concrete examples in the following section:

- “For example, prior to any interventions, compassionate community organizers, should develop knowledge and awareness that would assist them in identifying and mitigating gender inequities in compassionate community work, as well as knowledge and awareness of how neo-liberal discourse operates and can be resisted in compassionate community development (e.g., including the risk of over-emphasis on community resilience).”
- The paper would still benefit from more concrete examples of where or how gender inequities are reproduced or generated in comcoms, or how neo-liberal discourse negatively impacts comcoms or comcom outcomes. For the latter, now over-emphasis on community resilience is mentioned, but what is the negative outcome of such over-emphasis?

I invite the authors to reflect on the following question in their discussion: How should the ISCDP be interpreted and used? What do the authors suggest in terms of who these standards are really for? Are they to help communities increase sustainability, effectiveness and support? Should we use them instead to identify important conceptual or strategic gaps in existing guidance frameworks on Compassionate Communities? Or should they be used by researchers or other to categories initiatives or interventions as good or bad / successful or unsuccessful / counting as compassionate community initiatives or not? I doubt it’s the latter, however parts in the paper make me wonder about the framing. Below I’ll share an excerpt from the paper that sparked this reflection for me as a reader:

 “As such, the first step to develop compassionate communities is an effective strategy for community engagement 37 that includes community members from different backgrounds, to maximize the impact of civic participation in subsequent intervention development and implementation. For example, organizers should reflect on an engagement strategy to connect with women and caregivers, as well as broader community members.”

- Earlier in the paper, the authors suggest that ComCom initiatives should always be bottom-up endeavors, yet I can’t help but read these excerpts as having a top-down overtone. If comcoms organically develop bottom-up in response to local needs, they might not have an extensive or effective strategy for community engagement. Does this make them bad initiatives? or not comcoms? Would it not be more helpful or appropriate to fraise this in terms of what compassionate community initiatives should consider in order to connect well all segments of their community + in order to work sustainably and help those with the biggest needs? I realize it’s a minor shift in framing but I think an important one in order to align with the idea that comcoms are grassroots initiatives (OR, we should be explicit that they don’t need to be?)
- these feel like standards for organizations who want to implement something top-down such as researchers or governmental organizations”

We examine elements of the ISCDP to current conceptual and practice frames related to compassionate community development in Canada – specifically, to the Canadian Compassionate Community (CCC) Stages of Development 84 and Developing Compassionate Community (DCC) model 21.”

- Grammar issue here? Examine elements of the ISCDP *present in* current conceptual and practice frameworks?

Reviewer Expertise:

NA

I confirm that I have read this submission and believe that I have an appropriate level of expertise to confirm that it is of an acceptable scientific standard, however I have significant reservations, as outlined above.

# Reviewer report: Reviewer response for version 1

**DOI:** 10.21956/healthopenres.14698.r28650

**Published:** 2024-08-08

**Authors:** Steven Vanderstichelen

**Affiliations:** 1 Ghent University, Vrije Universiteit Brussel, Brussels, Belgium

Thank you for allowing me to review this interesting opinion article. I believe it raises important critical questions and the application of the ISCDP to the field is pertinent. The article is well written. Below I outline some problems I identified and questions I have for the authors that I hope they will address. Please excuse any potential bluntness in my comments and know that they are made with the intention to help the authors improve the manuscript.

 P3, 2nd par: “Canada’s compassionate community approach has been influenced by the Charter of Compassion and the Charter of Compassionate Cities ^17^.”

- This is factually wrong, Tompkins herself writes in the cited source that the compassionate cities charter is often confused with the charter for compassion in Canada, but does not say that the compassionate community approach in Canada has been inspired by it. Please correct this here.

Pg 4 Par 6: “In sum, despite the promise of compassionate community initiatives to enhance the quality of death through mutual support and inter-disciplinary collaboration, developing compassionate communities require mechanisms to promote meaningful participation and collaboration between stakeholders ^9,12,44,45^.”

- ComComs seek more than to enhance the quality of death. I agree with the other reviewer that the authors here adhere to an explicit service-oriented perspective and also leave bereaved people out of scope. Though I think small clarifications would help clear this up.

It would be helpful and increase the quality of the manuscript if the authors more explicitly showed, throughout the text, how the ISCDP address the critical issues identified by the authors at the beginning of the paper, i.e., reproduction of gender inequities (and I would argue of inequity in general), neo-liberal discourse associated with caring work, and over-emphasis on resilience.

 p7: “Though describing stages of compassionate community development, the model does not provide guidance about how to achieve bottom-up community participation and reduce the risk of reinforcing social inequalities in compassionate community development.”

- Would have liked the authors to comment on the latter at least, as they identify this as a key critical issue in the beginning of the paper (albeit focusing on gender iniquity specifically)

p7, figure 1: I’m not entirely convinced that it is possible to link each of the ISCDs to one single CC stage of development or even de CC actions described in Kelley’s model. While some clearly connect, the application of professional ethics and values in practice, for instance, surely applies to multiple stages. Additionally, promoting diversity and inclusion, building leadership and infrastructure, supporting learning for change also seem applicable the early stages of development. Also, I think a critical reflection on the proposed CCC stages of development is needed as well as they imply a linear process for the development of CCs. However, surely these can grow out of spontaneous small-scale collaborations for which, for instance, broader public awareness is not required, or there is no conscious strategic planning effort.  Finally, I think these stages are likely to be iterative and cyclical in nature, feeding into and triggering each other in nonlinear ways. We have argued previously that CCs are not simple, nor linear interventions and there is a danger in conceptualizing them as such. I don’t require the authors to include these references (they are publications I have (co-)authored), but for context:

- Vanderstichelen S, Deliens L. Chapter 24: Complexities and Challenges in a Public Health Palliative Care Research. Oxford textbook of public health palliative care. Oxford University Press, 2022.
- Bakelants H, Vanderstichelen S, et al. 2023. Researching Compassionate Communities: Identifying theoretical frameworks to evaluate the complex processes behind public health palliative care initiatives. Palliative Medicine, 37(2): 291-301. doi: 10.1177/02692163221146589

There’s a difference between what comcoms *ought* to be about, and what happens in practice. So far I think the authors appropriately highlight the risks of comcom models reproducing inequities (such as gendered inequities). Something that is not often discussed in mainstream public health palliative care literature.

 The contribution of the principles outlined by the ISCDP from the IACD are pertinent to the field of Public Health Palliative Care research and practice, in particular the development and study of Compassionate Community initiatives. However, it seems to me that EoL experiences and challenges are often avoided by people who could benefit from these initiatives and that it can be a challenge to get people to understand why they should care about EoL, PC, and bereavement in the first place. Is this not a strong barrier in our field? How can these principles help us address this? How do we get the community involved if they don’t want to be or they don’t understand why it is relevant to them? How do we ensure equitable involvement without forcing people.

 Some grammatical and punctuation issues throughout the manuscript should be attended to. A handful that I noticed:

- Page 4
  - Par 4: “when seeking to promoting” → when seeking to **promote**
- Page 5
  - “Community development practitioners thus commit to democratic participation, sustainable development and environmental justice, equality and human rights, social and economic justice, empowerment, and collectively ^54^.”
    - Should collectively be ‘collectivity’?
  - “This requires considerations of how and which the stakeholders are involved, as well as barriers and challenges to participation related to power structures ^59^.”
    - remove “the”
  - “In this regard, a community development approach to participatory planning should aim at 59:”
    - Reference is not in superscript
  - They can help identify and nurture local leadership and diverse skills and strengths in community members, and to help communities harness their community resources towards improvement61. To maximize change, however, practitioners should themselves build up good local, national, and international networks and partnerships ^61^.”
    - period missing between the two sentences

Reviewer Expertise:

NA

I confirm that I have read this submission and believe that I have an appropriate level of expertise to confirm that it is of an acceptable scientific standard, however I have significant reservations, as outlined above.

## References

[1] : Complexities and challenges in public health palliative care research.2022; 10.1093/med/9780198862994.003.0025 245-254 10.1093/med/9780198862994.003.0025

[2] : Researching Compassionate Communities: Identifying theoretical frameworks to evaluate the complex processes behind public health palliative care initiatives. *Palliat Med* .2023;37(2) : 10.1177/02692163221146589 291-301 10.1177/02692163221146589 36576313

# Response

**Published:** 2024-09-07

**Authors:** Wing-sun Chan

**Affiliations:** Division of Health Research, Lancaster University, Lancaster, England, UK

Comment: P3, 2nd par: “Canada’s compassionate community approach has been influenced by the Charter of Compassion and the Charter of Compassionate Cities17.”

 This is factually wrong, Tompkins herself writes in the cited source that the compassionate cities charter is often confused with the charter for compassion in Canada, but does not say that the compassionate community approach in Canada has been inspired by it. Please correct this here.

 Response: Thanks for this comment! I have revised this statement accordingly.  

 Comment: Pg 4 Par 6: “In sum, despite the promise of compassionate community initiatives to enhance the quality of death through mutual support and inter-disciplinary collaboration, developing compassionate communities require mechanisms to promote meaningful participation and collaboration between stakeholders9,12,44,45.”

 ComComs seek more than to enhance the quality of death. I agree with the other reviewer that the authors here adhere to an explicit service-oriented perspective and also leave bereaved people out of scope. Though I think small clarifications would help clear this up. Response: Thanks for your comment! I have revised line 1-2 in that paragraph to “to enhance the quality of death, mutual support, and civic participation…”  

 Comment: It would be helpful and increase the quality of the manuscript if the authors more explicitly showed, throughout the text, how the ISCDP address the critical issues identified by the authors at the beginning of the paper, i.e., reproduction of gender inequities (and I would argue of inequity in general), neo-liberal discourse associated with caring work, and over-emphasis on resilience.  

 Response: Thanks for your comment!  I added text into the first paragraph of the section on Insights from the International Standards for Community Development Practice for compassionate community development.  I have also added some text at the end of every ISCDP item to show explicitly how the ISCDP addresses the issues.  

 Comment: p7: “Though describing stages of compassionate community development, the model does not provide guidance about how to achieve bottom-up community participation and reduce the risk of reinforcing social inequalities in compassionate community development.”

 Would have liked the authors to comment on the latter at least, as they identify this as a key critical issue in the beginning of the paper (albeit focusing on gender iniquity specifically)   Response: Thanks for the comment. We have deleted the “does not” in the sentence as it was a typo.  

 Comment: p7, figure 1: I’m not entirely convinced that it is possible to link each of the ISCDs to one single CC stage of development or even de CC actions described in Kelley’s model. While some clearly connect, the application of professional ethics and values in practice, for instance, surely applies to multiple stages. Additionally, promoting diversity and inclusion, building leadership and infrastructure, supporting learning for change also seem applicable the early stages of development. Also, I think a critical reflection on the proposed CCC stages of development is needed as well as they imply a linear process for the development of CCs. However, surely these can grow out of spontaneous small-scale collaborations for which, for instance, broader public awareness is not required, or there is no conscious strategic planning effort.  Finally, I think these stages are likely to be iterative and cyclical in nature, feeding into and triggering each other in nonlinear ways. We have argued previously that CCs are not simple, nor linear interventions and there is a danger in conceptualising them as such. I don’t require the authors to include these references (they are publications I have (co-)authored), but for context:

 Vanderstichelen S, Deliens L. Chapter 24: Complexities and Challenges in a Public Health Palliative Care Research. Oxford textbook of public health palliative care. Oxford University Press, 2022. Bakelants H, Vanderstichelen S, et al. 2023. Researching Compassionate Communities: Identifying theoretical frameworks to evaluate the complex processes behind public health palliative care initiatives. Palliative Medicine, 37(2): 291-301. doi: 10.1177/02692163221146589 There’s a difference between what comcoms ought to be about, and what happens in practice. So far I think the authors appropriately highlight the risks of comcom models reproducing inequities (such as gendered inequities). Something that is not often discussed in mainstream public health palliative care literature.  

 Response: Thanks for the useful insights. Having considered the nonlinear trajectory CC development, I have dropped Figure 1 that juxtaposes the three models of compassionate communities and community development reflect values, stages, pre-conditions and activities, with implications for building value-driven and inclusive compassionate communities. The development trajectory is also iterative and cyclical.  

 Comment: The contribution of the principles outlined by the ISCDP from the IACD are pertinent to the field of Public Health Palliative Care research and practice, in particular the development and study of Compassionate Community initiatives. However, it seems to me that EoL experiences and challenges are often avoided by people who could benefit from these initiatives and that it can be a challenge to get people to understand why they should care about EoL, PC, and bereavement in the first place. Is this not a strong barrier in our field? How can these principles help us address this? How do we get the community involved if they don’t want to be or they don’t understand why it is relevant to them? How do we ensure equitable involvement without forcing people.  

 Response: Thanks for your comment! I agree it is a challenge and we have acknowledged the importance of community engagement in the second principle around engaging the community.    

 Comment: Some grammatical and punctuation issues throughout the manuscript should be attended to. A handful that I noticed:

 Page 4 Par 4: “when seeking to promoting” → when seeking to promote

 Response:  This has been revised.  

 Comment: Page 5 “Community development practitioners thus commit to democratic participation, sustainable development and environmental justice, equality and human rights, social and economic justice, empowerment, and collectively54.” Should collectively be ‘collectivity’?

 Response: This has been revised.  

 Comment: “This requires considerations of how and which the stakeholders are involved, as well as barriers and challenges to participation related to power structures59.” remove “the”   Response: This has been revised.  

 Comment: “In this regard, a community development approach to participatory planning should aim at 59:” Reference is not in superscript

 Response: This has been revised. The reference format will be fixed by the editorial team. 

 Comment: They can help identify and nurture local leadership and diverse skills and strengths in community members, and to help communities harness their community resources towards improvement61. To maximize change, however, practitioners should themselves build up good local, national, and international networks and partnerships61.” period missing between the two sentences  

 Response:  This has been revised.  

# Reviewer report: Reviewer response for version 1

**DOI:** 10.21956/healthopenres.14698.r28599

**Published:** 2024-07-22

**Authors:** Julian Abel

**Affiliations:** 1 Compassionate Communities UK, St. Martin, Helston, UK

I completely agree with the author about the need for knowledge, skills and practice methods relating to compassionate communities. Too often, health care practitioners make assumptions about compassionate communities without these knowledge or skills and the impact of these initiatives is often limited and short lived. Worse still, they can have a negative impact on the 3 middle action points of the Ottawa Charter for Health Promotion, namely weakening supportive environments in the community, weakening community action and decreasing personal skills. However, the author has misunderstood the essence of public health palliative care, and compassionate communities in particular. It is such a shame because the points he makes about the IACD and the standards for practice are highly relevant. Importantly, cultural respect is fundamental to compassionate communities, imbued with curiosity and humility to overcome Eurocentric values and assumptions. Diversity should be welcomed and appreciated as a richness, and often the health service perspective dominates community cultural practice and values. 

 Three fundamental conceptual flaws concerning compassionate communities at end of life are made by the author.

 The first is the focus on home death. This obsession with home death is a health service perspective. The experiences of death, dying, loss and care giving can go on for months or years, and in particular, loss can last a lifetime. Public health palliative care focusses on all of these experiences. Death, dying, loss and care giving involves everyone, as these experiences are repeated multiple times until our own final one. In addition, the support given is in the multiple environments spoken about in the compassionate city charter of Allan Kellehear. Simply focusing on  home death as an outcome misses the major places of intervention of the broader aspects of public health palliative care. 

 The second major conceptual problem is that of the focus on resilience as being a major outcome of compassionate communities. Resilience is an unfortunate term that suggests that we have to put up with what is going on. However, in addition to sadness and loss, there is love, friendship, courage, intimacy, celebration, ritual, meaning making, healing, deepening of relationships that can last for a whole lifetime and other qualities of richness that are found mixed in with the other experiences of suffering. Resilience falls way short in capturing this. 

 The third conceptual problem concerning compassionate communities in this article is that the focus is on the dying person. However, as with point 2, the qualities of relationships of people engaged in the supportive network is as important as the care given to the dying person. We live in the context of relationships, expressed beautifully in the philosophy and practice of Ubuntu, mentioned in The Lancet Commission on the Value of Death. What happens between members of the supportive network is a critical part of compassionate communities and is fundamental to health and well being. Please see the chapter in The Oxford Textbook of Public Health Palliative Care on this matter. The author assumes that the main focus of compassionate communities is caring work done primarily by females. It is true that much of the caring work is done by females but from a  compassionate community perspective, hands on caring is one aspect of a plethora of experiences and tasks. In addition to caring, there is friendship and other qualities mentioned above. There are ritual, lifts, dog walking, shopping, cooking, cleaning and a whole variety of other activities that are part of compassionate communities. Gender inequity is an aspect and this happens in a variety of ways, not just male to female or vice versa. There are many forms of inequity, including those of diversity. 

 My suggestion would be to rewrite the introductory section of this article. What has happened is something of a straw man argument, where resilience is used as a basis of a conceptual framework and then this is shot down in the context of compassionate communities. 

 There are a couple of minor details which need amending. Bonnie Tompkins and Pallium were not inspired by the Charter for Compassion as the basis of their work. This is a separate movement. Rather, it is public health palliative care, including compassionate communities and the Compassionate City Charter based on the new public health methods of participatory community development, prevention, harm reduction and early intervention. 

 The article refers to health service access in the context of inequity. From the perspective of public health palliative care, inequity of access to health services is not the main issue. As seen with the Kelley Model, it is not just inequity of access. Many of the services provided are not appropriate or even wanted from diverse cultural perspectives. What is needed is health service reorientation, as specified by point 5 of the Ottawa Charter. If these is humility and curiosity, the basis of relationship forming which is key to the practices of participatory community development, then community strengths can be discovered and health services adapt to the wishes and desires of the community, rather than thinking one size fits all and that palliative care provision is the gold standard.

Reviewer Expertise:

Public health palliative care

I confirm that I have read this submission and believe that I have an appropriate level of expertise to state that I do not consider it to be of an acceptable scientific standard, for reasons outlined above.

## References

[1] Oxford Textbook of Public Health Palliative Care.2022; 10.1093/med/9780198862994.001.0001 10.1093/med/9780198862994.001.0001

# Response

**Published:** 2024-09-07

**Authors:** Wing-sun Chan

**Affiliations:** Division of Health Research, Lancaster University, Lancaster, England, UK

I completely agree with the author about the need for knowledge, skills and practice methods relating to compassionate communities. Too often, health care practitioners make assumptions about compassionate communities without these knowledge or skills and the impact of these initiatives is often limited and short lived. Worse still, they can have a negative impact on the 3 middle action points of the Ottawa Charter for Health Promotion, namely weakening supportive environments in the community, weakening community action and decreasing personal skills. However, the author has misunderstood the essence of public health palliative care, and compassionate communities in particular. It is such a shame because the points he makes about the IACD and the standards for practice are highly relevant. Importantly, cultural respect is fundamental to compassionate communities, imbued with curiosity and humility to overcome Eurocentric values and assumptions. Diversity should be welcomed and appreciated as a richness, and often the health service perspective dominates community cultural practice and values. 

 Three fundamental conceptual flaws concerning compassionate communities at end of life are made by the author.

**Comment: **The first is the focus on home death. This obsession with home death is a health service perspective. The experiences of death, dying, loss and care giving can go on for months or years, and in particular, loss can last a lifetime. Public health palliative care focusses on all of these experiences. Death, dying, loss and care giving involves everyone, as these experiences are repeated multiple times until our own final one. In addition, the support given is in the multiple environments spoken about in the compassionate city charter of Allan Kellehear. Simply focusing on  home death as an outcome misses the major places of intervention of the broader aspects of public health palliative care. 

**Response: **Thank you for useful comment! We realize that home death is typically more of a healthcare service issue; indeed, our dying at home project did not set out as a study of compassionate communities (although we did interview some compassionate community advocates as one of several types of stakeholders, and many of them emphasized home deaths as ideal). Our goal in this paper was to reflect on and transfer some of our findings’ potential relevance to the compassionate communities movement. In the second paragraph (“relevant research”), we have clarified our understanding about compassionate communities - as not a healthcare service coverage issue but rather an issue of social structural transformation. In that section, we identify three potential challenges for compassionate community development (i.e., gender inequalities, the neo-liberal discourse associated with caring work, and an over-emphasis on community resilience) based on our experience in the dying at home in Canada research project; for us, this is just a starting point to underscore the importance of community development principles.

**Comment:** The second major conceptual problem is that of the focus on resilience as being a major outcome of compassionate communities. Resilience is an unfortunate term that suggests that we have to put up with what is going on. However, in addition to sadness and loss, there is love, friendship, courage, intimacy, celebration, ritual, meaning making, healing, deepening of relationships that can last for a whole lifetime and other qualities of richness that are found mixed in with the other experiences of suffering. Resilience falls way short in capturing this. 

**Response: **In the seventh paragraph of the section covering relevant research, we have revised the text and clearly stated that “ Importantly, a compassionate community does not seek to develop community resilience (i.e., the deficit model) in facing death, dying, and grieving, but focuses on linking dying persons and their families to the community ecology (i.e., the asset model) and on recognizing and developing more nuanced and complex experiences of suffering.” We agree, in fact, that resilience is an inadequate conceptualization for compassionate communities; what we highlight is the risk that the concept can be interpreted and implemented in this narrow way, particularly in a political-economic context of neoliberalism.

**Comment: **The third conceptual problem concerning compassionate communities in this article is that the focus is on the dying person. However, as with point 2, the qualities of relationships of people engaged in the supportive network is as important as the care given to the dying person. We live in the context of relationships, expressed beautifully in the philosophy and practice of Ubuntu, mentioned in The Lancet Commission on the Value of Death. What happens between members of the supportive network is a critical part of compassionate communities and is fundamental to health and well being. Please see the chapter in The Oxford Textbook of Public Health Palliative Care on this matter. The author assumes that the main focus of compassionate communities is caring work done primarily by females. It is true that much of the caring work is done by females but from a  compassionate community perspective, hands on caring is one aspect of a plethora of experiences and tasks. In addition to caring, there is friendship and other qualities mentioned above. There are ritual, lifts, dog walking, shopping, cooking, cleaning and a whole variety of other activities that are part of compassionate communities. Gender inequity is an aspect and this happens in a variety of ways, not just male to female or vice versa. There are many forms of inequity, including those of diversity. 

**Response: **Thank you for your comment. We revised how we cover this in the ‘relevant research’ section. We have revised the text in the first paragraph of that section to emphasise that the focus of compassionate communities is on social structural transformation, and have sought to clarify this elsewhere in the paper as well. In addition, we have  reworked some of our discussion of caring to explain that not only more intensive hands on care involvement for dying persons tends to be gendered, but that women are also often responsible for a vast majority of other less tangible forms of care in their communities such as relational and emotional work, kinkeeping, social connections, and the like.

**Comment:** My suggestion would be to rewrite the introductory section of this article. What has happened is something of a straw man argument, where resilience is used as a basis of a conceptual framework and then this is shot down in the context of compassionate communities. 

 There are a couple of minor details which need amending. Bonnie Tompkins and Pallium were not inspired by the Charter for Compassion as the basis of their work. This is a separate movement. Rather, it is public health palliative care, including compassionate communities and the Compassionate City Charter based on the new public health methods of participatory community development, prevention, harm reduction and early intervention. 

**Response:** Thank you for catching this error! We have revised the introduction section accordingly.

**Comment:** The article refers to health service access in the context of inequity. From the perspective of public health palliative care, inequity of access to health services is not the main issue. As seen with the Kelley Model, it is not just inequity of access. Many of the services provided are not appropriate or even wanted from diverse cultural perspectives. What is needed is health service reorientation, as specified by point 5 of the Ottawa Charter. If these is humility and curiosity, the basis of relationship forming which is key to the practices of participatory community development, then community strengths can be discovered and health services adapt to the wishes and desires of the community, rather than thinking one size fits all and that palliative care provision is the gold standard.

**Response:** Thanks for your comment! We have revised the text accordingly in the second paragraph of the introduction section.
